# Subcellular targeting of survivin peptides: A multi-axis approach to cancer therapy

**DOI:** 10.1016/j.omton.2026.201145

**Published:** 2026-02-17

**Authors:** Anup Singh Pathania

**Affiliations:** 1 Department of Pharmacology and Experimental Neuroscience, University of Nebraska Medical Center, Omaha, NE 68198, USA

## Main text

Survivin is a highly attractive cancer target because it is strongly overexpressed in many tumors but largely absent in most normal adult tissues, providing relative tumor selectivity.^1^ As an inhibitor of apoptosis protein (IAP), survivin binds and sequesters the mitochondrial pro-apoptotic factor SMAC/Diablo, which normally neutralizes IAPs such as XIAP, allowing caspase-3 and caspase-9 to drive apoptosis.^2^^,^^3^ By trapping SMAC/Diablo, survivin preserves IAP function, keeps caspases inactive, and supports cancer cell survival.^3^ This makes the survivin-SMAC/Diablo interface a key survival checkpoint and a logical target for pro-apoptotic therapies.

Building on this, in this issue of *Molecular Therapy Oncology*, Santhanam and colleagues identified a previously unrecognized SMAC-binding sequence within survivin and used it to design a 25-amino-acid survivin-derived peptide (1H13) spanning several critical functional regions.^4^ When converted into stabilized, cell-penetrating forms targeted to specific cellular compartments, these peptides disrupt survivin dimerization and its interactions with partner proteins. This blocks survivin anti-apoptotic activity and inhibits proliferation across multiple human cancer cell lines.

Survivin-targeted therapeutics remain largely experimental, with no Food and Drug Administration-approved drugs, yet they hold significant promise across multiple modalities. Small-molecule inhibitors, such as YM155, suppress survivin transcription and have advanced to phase 2 trials in breast, lung, melanoma, and prostate cancers. While they enhanced chemotherapy effects, their development faced setbacks due to toxicity and modest efficacy.^5^ Other compounds like FL118 (multi-IAP transcriptional suppression), terameprocol (survivin transcription inhibition), and shepherdin (Hsp90-survivin disruption) impair survivin function in preclinical models, although none are fully survivin specific.^6^

Peptide-based survivin vaccines, including SurVaxM and survivin 2B, face significant limitations.^7^^,^^8^ Survivin is poorly immunogenic; short peptides are rapidly degraded, bind weakly to major histocompatibility complex, and often induce tolerance rather than robust immunity. Human leukocyte antigen restriction and tumor heterogeneity further limit applicability and enable immune escape. Peptides also have short half-lives and face delivery challenges, while immunosuppressive tumor microenvironments blunt responses.^9^ Clinical trials have shown modest efficacy, variable patient outcomes, and occasional off-target effects.

The 1H13 peptide addresses several of these limitations.^4^ It is engineered for stability (D-amino acids), targeted delivery (nucleus/mitochondria), controllable dosing, and direct on-target effects measurable within days. The peptide spans survivin residues 83–106, a multifunctional “hotspot” that simultaneously disrupts four essential survivin functions: dimerization (via Leu98/Phe101), SMAC binding (freeing pro-apoptotic caspases), tubulin/microtubule interactions (destabilizing mitotic spindles), and chromosomal passenger complex/nuclear import signals (blocking centromere recruitment). This multi-hit strategy makes resistance via single mutations highly unlikely, as altering one residue would compromise all four activities and likely disrupt survivin folding and function. The effect is analogous to severing multiple load-bearing pillars of a bridge, ensuring total collapse rather than easy repair.

Survivin localizes to distinct cellular compartments, including cytosol, mitochondria, and nucleus, where it executes compartment-specific survival functions. In the cytosol, it inhibits caspase activation; in the mitochondria, it regulates SMAC/Diablo-mediated death signaling and maintains mitochondrial integrity; and in the nucleus, it controls mitosis via the chromosomal passenger complex. To exploit these locations, the authors engineered compartment-targeted 1H13 peptides: cytosol targeted (*Drosophila* antennapedia sequence, ANTP), mitochondria targeted (D-Arg-Dmt-Orn-Phe-NH2), and nucleus targeted (RrRK motif). Some peptides replaced natural L-amino acids with D-enantiomers to improve stability. Cytosol- and mitochondria-targeted peptides primarily disrupt anti-apoptotic functions, whereas nuclear targeting mainly impairs mitotic/proliferative roles, secondarily triggering apoptosis via failed cell division.

All organelle-targeted 1H13 peptides exhibit robust cytotoxicity across diverse cancer cell lines, although efficacy varies with targeting sequence and D/L composition. Replacing L-amino acids with D-amino acids reduces cell death, particularly when all residues are converted to the D-form. Cytosol-targeted peptides with all L-amino acids or partial D-substitutions (2/3D) retain strong cytotoxic activity (∼90% cell death), whereas fully D-amino acid versions show markedly reduced activity (∼30%). Similarly, mitochondria- and nucleus-targeted peptides with partial D-substitutions perform comparably or slightly worse than all-L peptides, while fully D-modified versions show reduced binding and lower maximal cell death. This demonstrates that the native L-amino acid configuration is critical for peptide activity and excessive D-amino acid substitution compromises efficacy.

*In vivo*, 1H13 peptides targeted to the cytosol, mitochondria, or nucleus markedly inhibited the growth of A549 lung cancer xenografts in mice. Treatment strongly decreased proliferation (Ki-67) and increased apoptosis (TUNEL-positive DNA fragmentation); reduced survivin, SMAC, and tubulin levels; and increased p53. Tumor volume and weight decreased by up to >90%, with nuclear 2/3D-1H13 being most effective. Nuclear localization likely delivers a superior “functional hit” on pathways driving proliferation and survival. Partial D-amino acid substitution (2/3D) enhances stability against degradation while preserving binding to survivin, yielding sustained and potent inhibition at a highly sensitive subcellular site.

Limitations include reliance on a single tumor type (A549 lung cancer) grown as a subcutaneous xenograft in nude mice, which does not fully recapitulate the human tumor microenvironment, metastasis, or immune interactions. Peptides were administered intravenously on a fixed schedule, without dose response, pharmacokinetic, or toxicity studies in normal organs, leaving optimal dosing and safety uncharacterized.

Interestingly, peptides increased CD8^+^ T cell and natural killer cell infiltration in these xenografts, while upregulating PD-1/PD-L1 on tumor and host cells, suggesting potential responsiveness to checkpoint blockade. Although nude mice are immunocompromised due to the absence of a functional thymus and reduced conventional T cells, they retain partial immune functionality. These observations, therefore, provide provisional insights into immunomodulatory effects, which may differ substantially in patients.

Overall, this study demonstrates that directing the same survivin-derived peptide to different organelles produces strong, but distinct, anti-tumor effects, highlighting subcellular targeting as a powerful design principle. High survivin expression correlates with advanced stage, metastasis, recurrence, and shorter survival in many cancers, including lung cancer.^10^ A single survivin-directed construct can induce mitotic catastrophe, apoptosis, metabolic changes, and immune modulation, exemplifying a shift from “single-pathway” drugs to agents that simultaneously hit multiple cancer hallmarks.

Importantly, the work shows that a rationally designed peptide can disrupt a specific protein-protein interaction (survivin dimerization and complexes) and generate strong therapeutic effects. This is broadly relevant for targeting “undruggable” cancer drivers that function through multiprotein complexes rather than enzymatically active sites.

## Declaration of interests

The author declares no competing interests.
